# The impact of human mobility data scales and processing on movement predictability

**DOI:** 10.1038/s41598-021-94102-x

**Published:** 2021-07-26

**Authors:** Kamil Smolak, Katarzyna Siła-Nowicka, Jean-Charles Delvenne, Michał Wierzbiński, Witold Rohm

**Affiliations:** 1 Institute of Geodesy and Geoinformatics, Wrocław University of Environmental of Life Sciences, Wrocław, Poland; 2 Urban Big Data Centre, University of Glasgow, Glasgow, UK; 3 School of Environment, The University of Auckland, Auckland, New Zealand; 4 Institute of Communication Technologies, Electronics, and Applied Mathematics, Université Catholique de Louvain, Louvain-la-Neuve, Belgium; 5 Spyrosoft S.A., Kraków, Poland

**Keywords:** Computational science, Statistics

## Abstract

Predictability of human movement is a theoretical upper bound for the accuracy of movement prediction models, which serves as a reference value showing how regular a dataset is and to what extent mobility can be predicted. Over the years, the predictability of various human mobility datasets was found to vary when estimated for differently processed datasets. Although attempts at the explanation of this variability have been made, the extent of these experiments was limited. In this study, we use high-precision movement trajectories of individuals to analyse how the way we represent the movement impacts its predictability and thus, the outcomes of analyses made on these data. We adopt a number of methods used in the last 11 years of research on human mobility and apply them to a wide range of spatio-temporal data scales, thoroughly analysing changes in predictability and produced data. We find that spatio-temporal resolution and data processing methods have a large impact on the predictability as well as geometrical and numerical properties of human mobility data, and we present their nonlinear dependencies.

## Introduction

The proliferation of mobile devices has a significant impact on studying human mobility in areas such as disease spread modelling^1^, utility management^2^, and urban planning^3^. The majority of these applications is based on the regularity and predictability of the movement of individuals^4^, helping to understand the human behaviour underlying mobility. This raised questions of how predictable individual movement trajectories are and what impacts their predictability.

In 2010, Song et al.^5^ adopted an entropy measure to quantify the predictability of individual mobility using a mobile phone location dataset collected from 45,000 users. Their locations were assigned to a currently connected cell tower and grouped into 1-h time intervals (called time-bins), creating individual movement trajectories. Each of them consisted of a sequence of symbols where each symbol corresponded to the tower. The proposed method calculates the entropy using a Lempel-Ziv data compression algorithm^6^ which enables measuring the probability of finding a particular time-ordered subsequence in the trajectory. Then, the predictability measure is derived from the calculated entropy by solving a limiting case of Fano’s inequality (originally related to the information decrease in the message obtained over a noisy channel)^7^. Their work reported the upper bound of human mobility predictability to be $\Pi _{max} = 93\%$.

This aforementioned work was followed by numerous studies investigating the predictability of human mobility which used data gathered from different populations and at various spatio-temporal scales (but not limited to human mobility only, as other types of sequences, such as vehicle movement and radio spectrum state, were evaluated^8–10^). These studies derived different upper bounds of predictability ranging from 43% up to 95%^11^. Lu et al.^4,12^ applied a predictability estimation algorithm to two different mobile phone datasets. In the first study, the data were spatially assigned to cell towers, thus had a similar spatial resolution to the data used in the work of Song et al., but they had a low (daily) temporal resolution, which resulted in a different $\Pi _{max} = 85\%$. In the second work, the movement of individuals was recorded at a low spatial resolution of a few square kilometres on average, which yielded yet another result of $\Pi _{max} = 88\%$. The impact of the spatio-temporal resolution of the movement trajectories was noted in later works using high-precision data gathered through Global Navigation Satellite Systems (GNSS) loggers^11,13–17^. A decrease in spatial resolution caused an increase in predictability, hence precise GNSS data were less predictable in their raw form. On the other hand, when the temporal resolution of the data decreased, the predictability was also falling.

The key part of all predictability studies, also important for human mobility data mining, is converting the original data source into a sequence of visited locations. Multiple studies^14–16^ demonstrated that not only the spatio-temporal resolution of the data but also the trajectory processing method significantly affects the entropy and predictability of the data. An original approach from the work of Song et al.^5^ was to determine a person’s location at regular time intervals $\Delta t$ (we refer to it as the *next time-bin approach*). Ikanovic and Mollgaard^15^, and Cuttone et al.^16^ simultaneously noticed that this method creates many self-transitions, that is cases when a person stays in the same location in the next time-bin. Because predictability is measured as the number of correctly predicted symbols, the high number of self-transitions artificially enhances its value as it is easy to predict them. Moreover, movement trajectories have data gaps (i.e. data are missing because the device stopped working or the position was recorded with a high error and had to be removed^18^), thus this method tends to create empty time-bins. These missing data decrease predictability estimation. To address it, Ikanovic and Mollgaard^15^, and Cuttone et al.^16^ have proposed an alternative approach, referred to as the *next place sequence*, where all self-transitions are eliminated, also removing the temporal dimension from the sequences. For the same sequence processed with the next place approach the predictability was lower (dropping from $\Pi _{max} = 95\%$ to around $\Pi _{max} = 70\%$).

High-precision location data require the application of spatio-temporal aggregation methods to assign spatially close (lying within a predefined range) data points to a single location, called stay-region. In human mobility studies, this is a commonly applied data processing step that is designed to group data points into meaningful locations which form a sequence^19,20^. In the mobile phone data, this issue is usually omitted as mobile phone locations are recorded as cell tower locations. The movement is recorded as a sequence of towers’ identifiers, and the area tessellation is based on the Voronoi diagram^5^. In the majority of works on human mobility predictability, for the next time-bin approach, space was divided into cells of a uniform grid^14–17^. Each data record was assigned to the cell of the grid within which it lied and was represented by a unique cell identifier. Then, the data were temporally aggregated by selecting a location within which a person spent most of the time during the current time interval. Different sizes of cells were used to assess the impact of data resolution on predictability. Another method of spatial aggregation was used for the next place sequences^15,16^, where points were aggregated in a two-step clustering process. First, data were filtered to remove noisy data points and then the remaining locations were grouped into clusters based on spatial and/or temporal conditions, ensuring their similarity, thus describing the same location. Data filtering is motivated by removing meaningless data points such as travels and stops in traffic jams to focus only on the intentionally visited places. Temporal aggregation does not apply to this case.

Movement trajectories processing methods have been found to introduce variations into the movement sequences^11,15,16,21^, which means that the observed mobility depends on the method used to process the data. Therefore, not only entropy is affected but other statistical measures of mobility are also modified. This effect is not limited to predictability studies as processing movement trajectories into sequences is a widely used approach in mobility data mining^20^, hence it spreads on a large portion of mobility studies. The variability of mobility measures is related to the problem of statistical bias arising from the spatial aggregation of point-based measures and was identified almost 90 years ago^22^ and is known as a modifiable areal unit problem (MAUP). The same statistical bias being a result of a modification of the temporal dimension, conceptualised later by Çöltekin et al.^23^ was named modifiable temporal unit problem (MTUP). The recent research by Alessandretti, Aslak and Lehmann^24^ investigates the issue of spatio-temporal scales at which we quantify individual movement trajectories. A large portion of the literature, majorly originating from a physics-based point of view on empirical analyses of human mobility, describes human movement trajectories as scale-free^25–27^. However, this recent study shows that human mobility is characterised by nested containers at different spatial levels. These containers are corresponding to the spatial scales of human mobility at which we can quantify the movement. The impact of temporal data resolution has also been investigated in mobility studies^28^, which resulted in finding its strong impact on commonly used mobility indicators, such as the number of daily trips. However, such analyses have not yet been done in human predictability studies. The same individual movement trajectories observed at different scales and sampled at diverse temporal intervals are described by various sequences and hence, yield different values of predictability.

Until now, high-resolution GNSS data were used to investigate the relationship between data resolution and predictability. However, in these cases, the impact of this relationship was shown only for the next time-bin approach using grid-based aggregation and at a limited set of scales, from a hundred to a few hundred metres spatially and from 5 min up to 2 h temporally. We fill this gap by thoroughly studying the effect of spatio-temporal aggregation methods for the next time-bin and the next place approaches across a range of scales, spanning from fine spatio-temporal resolutions of ten metres and 5 min up to the data maximum extent. In addition to the grid-based aggregation, we also verify the impact of clustering on the created sequences. For clustering, we use the density-based spatial clustering of applications with noise (DBSCAN) which was predominantly used in predictability studies to create the next place sequences^15,16^. In contrast to the grid-based approach, clustering parameters cannot be directly related to the spatio-temporal magnitude of aggregation, impeding the selection of their correct values. Moreover, as the methodology proposed by Song et al.^5^ was extensively followed by the human mobility research community, researchers found entropy estimates from missing data, calculated using the original method (denoted here as $\hat{H}_{shuff}$), being inaccurate and proposed two alternative methods. We evaluate these methods at the mentioned levels of temporal resolution.

## Results

### Human mobility dataset

To ensure a precise depiction of human mobility at all spatial and temporal scales, we use high-resolution individual movement trajectories collected from mobile devices of people living in London, UK. The data were harvested through smartphone applications, where the location of devices was determined using GNSS receivers installed in mobile phones. Following the methodology of Song et al.^5^ we select trajectories using a fraction of missing records in hour-long intervals *q* and a trajectory length *d*, expressed in days, as filtration criteria. However, in comparison to this work, we decided to use more strict criteria to minimise the impact of missing data and short movement trajectories on results. We consider only the trajectories with $q \le 0.15$ and $d \ge 28$ consecutive days. From the initial dataset consisting of nearly five million devices, after the filtration, from trajectories fulfilling the above criteria, we randomly select 500 people. As a result, the median fraction of missing records in the dataset is $q = 0.04$ and the length of movement trajectories vary from 28 to 31 days. The selected dataset consists of almost five million data points.

We start with data processing for which details can be found in the Methods section. Due to the high temporal resolution of the data, movement trajectories have to be filtered, as a large portion of data points was recorded during travels between locations which from the perspective of mobility prediction are unimportant. Some stops, such as those caused by traffic jams, are also meaningless for mobility prediction and should be discarded during the process. For that, we apply a commonly used approach for stationary points (stay-points) detection which also accounts for GNSS positioning error^19^. This creates sequences of detected stationary points which will be later aggregated into the sequences of stay-regions. Although this step was not applied before the grid-based clustering in existing predictability studies, we argue that it should be done in all cases due to the reasons mentioned above.

### Entropy estimation from missing data

The next time-bin approach forces sequences to be indexed by temporally ordered time intervals $\Delta t$ which in turn may create situations where time-bins are empty. For example, if $\Delta t = 1\,\text {h}$ and an individual does not record any positions for one whole hour, the resulting sequence will be empty at this specific interval. Record completeness is measured by the fraction of empty records *q*, originally expressed for $\Delta t = 1\,\text {h}$. Here, we measure the mean *q* for every calculated sequence for a wide range of $\Delta t$ from 5 min up to 12 days. For higher values of $\Delta t$, the value of *q* is lower. For resolutions lower than 1 h, no empty records are present in the next time-bin sequences. Results are presented in Table 1.

Three different approaches to entropy estimation from missing data were presented across the literature. These include the initial work^5^, and the two following studies^15,17^. We denote those methods as $\hat{H}_{shuff}$, $\hat{H}_{\Delta e}$, $\hat{H}_{unc}$, respectively. To verify their accuracy, for each method we calculate the average error of entropy estimation and present them in Table 1. For the experiment, we use 100 complete movement sequences for which we calculate the real entropy serving as a reference value. Although, it was concluded that for the sequences with $q < 0.25$ estimated actual entropy can be considered equal to the real entropy^17^, we select an even more strict threshold selecting users with $q < 0.15$ for the entropy estimation methods evaluation. After that, we gradually remove records to simulate missing data up to the level of $q \le 0.6$, estimate actual entropy and compare it with a reference value to assess the methods. Details of these methods and our experiment design can be found in the Methods section.

**Table 1:** Comparison of average actual entropy estimation errors from incomplete mobility sequences for $q \le 0.60$ using three approaches $\hat{H}_{shuff}$, $\hat{H}_{\Delta e}$, $\hat{H}_{unc}$. Estimations are compared to the real entropy *H* calculated on complete movement sequences and at different levels of temporal aggregation $\Delta t$. *q* is an average fraction of missing data in movement trajectories used in the experiment. A 95% confidence interval across the sequences is presented next to the average values of measures.

$\Delta t$	*q* (%)	$\hat{H}_{shuff}/H$ (%)	$\hat{H}_{\Delta e}/H$ (%)	$\hat{H}_{unc}/H$ (%)
5 min	$5.2 \pm 0.2$	$50.6 \pm 4.8$	$3.0 \pm 0.6$	$43.2 \pm 2.1$
10 min	$4.2 \pm 0.2$	$40.2 \pm 4.4$	$3.5 \pm 0.9$	$23.9 \pm 1.5$
30 min	$2.0 \pm 0.1$	$26.5 \pm 1.7$	$4.5 \pm 1.4$	$19.6 \pm 1.3$
1 h	$0.7 \pm 0.6$	$25.0 \pm 1.8$	$5.7 \pm 1.9$	$19.4 \pm 1.3$

The best performing method is $\hat{H}_{\Delta e}$ with the lowest error across various $\Delta t$. Interestingly, the error of $\hat{H}_{\Delta e}$ is growing with $\Delta t$ which is not observable in the remaining methods. The original method $\hat{H}_{shuff}$ returns relatively high errors, while $\hat{H}_{unc}$ is performing slightly better. As all of these methods are based on an estimation of functional dependency between entropy calculated on complete and incomplete data, we study the type of this relationship. We assume that function fits well to the estimated ratio when the coefficient of determination $R^2$ > 0.9. We find that relationship in $\hat{H}_{\Delta e}$ and $\hat{H}_{unc}$ can always be estimated by an offset exponential function, while in the case of $\hat{H}_{shuff}$ it is not always the case as some relationships are linear. Moreover, the number of users with linear relationships between estimated entropies increases together with $\Delta t$.

### Full-scale predictability estimation

We now focus on the predictability of individual mobility sequences obtained via different methods at various spatio-temporal scales. We separately evaluate the impact of spatial and temporal resolutions on the three types of predictability.

#### Impact of spatial aggregation on predictability estimation

**Figure 1:**
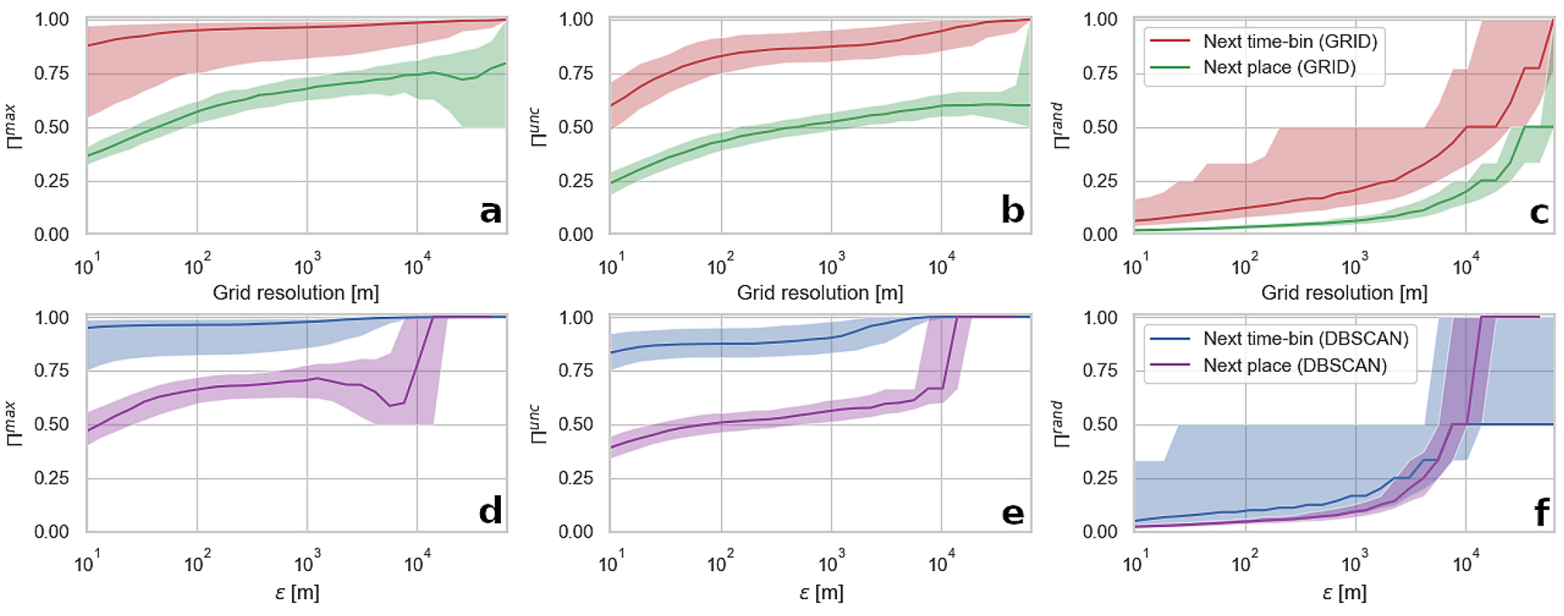
An impact of parameters (grid resolution and $\epsilon$ parameter of the DBSCAN algorithm) controlling the spatial resolution of aggregated data on three types of predictability (actual (**a**, **d**), uncorrelated (**b**, **e**) and random (**c**, **f**)) for two types of mobility sequences and data aggregation methods (in rows: GRID (**a**–**c**) and DBSCAN (**d**–**f**)). The lines represent the median value and the shaded area indicate the interquartile range of predictability measures calculated for all the users in the dataset.

Figure 1 presents the change of actual predictability ($\Pi ^{max}$), uncorrelated predictability ($\Pi ^{unc}$), and random predictability ($\Pi ^{rand}$) (see Methods for details on these measures), with parameters used for spatial data aggregation. $\Pi ^{max}$ for the next time-bin approach is high across the whole range, starting from 87.6% for grid-based approach and 94.8% for DBSCAN at a high spatial granularity of the data and reaching up to 99.8% and 99.9%, respectively. For the whole range of spatial parameters, the data processed with DBSCAN are more predictable. The spread of predictability among users is relatively high (14.5% for DBSCAN and 17.6% for grid-based approach, measured by the interquartile range (IQR)) and drops with the increase in data spatial aggregation. The dependence of $\Pi ^{max}$ on spatial parameters in the next time-bin approach follows an exponential function with $R^2 > 0.88$. In the case of the next place sequences, the values of $\Pi ^{max}$ are much lower and start from 46.7% for DBSCAN and 36.3% for the grid-based approach. Then predictability slowly grows reaching over 70% in both methods when the aggregation parameter is around $10^3$ metres. Up to this point, clustered sequences are more predictable than the data processed with the grid-based approach. Over that level the $\Pi ^{max}$ in the grid-based aggregation still grows, reaching 79.5% for the maximum aggregation level, while in the clustering-based method the predictability first drops to reach maximum value for the higher aggregation levels. These fluctuations are caused by the length of the sequences. Clusters created by the DBSCAN method are larger (see Fig. 3(a)), which in the case of the next place approach makes sequences very short. For example, when an aggregation level is high, all the stationary points are assigned to a single stay-region. This in turn results in sequences consisting of the same symbol, repeated multiple times, which are then truncated to the length of one to remove all redundant symbols. By definition, such a sequence is completely predictable. In another case, when two stay-regions are detected, sequence predictability is 50% because a user can be found in one of these positions with an equal probability. The spread of $\Pi ^{max}$ is lower in the case of the next place sequences (14.4% for DBSCAN and 11.5% for grid-based approach, measured by IQR).

Values of $\Pi ^{unc}$ are also higher for the next time-bin sequences and similarly to $\Pi ^{max}$, the sequences processed with DBSCAN are more predictable. Interestingly, $\Pi ^{unc}$ for the next place sequences is close to $\Pi ^{max}$ for the low magnitude of spatial aggregation. Values of $\Pi ^{unc}$ are following an exponential function with $R^2 > 0.89$. $\Pi ^{rand}$, which is based on the number of unique locations present in the sequence, is similar for the next time-bin and the next place sequences and is lower for the DBSCAN algorithm. Values of $\Pi ^{rand}$ are following an exponential function with $R^2 > 0.91$.

#### Impact of temporal aggregation on predictability estimation

The next time-bin sequences can also be analysed for variations at different temporal resolutions $\Delta t$. We find the relationship between the estimated predictability values and $\Delta t$ being irregular. Values of $\Pi ^{max}$ are decreasing with increasing $\Delta t$ up to a resolution of one day for DBSCAN and six days for grid-based aggregation. After, they rise to 99.9% when trajectories consist of the same symbol, repeated multiple times, which is the most often visited location. Interestingly, the spread of all actual predictability values is increasing with $\Delta t$. Similarly to $\Pi ^{max}$, values of $\Pi ^{unc}$ also fall along with $\Delta t$ rise but this decrease is lower than in the case of $\Pi ^{max}$. $\Pi ^{rand}$ shows reversed dependency growing with $\Delta t$ increase.

**Figure 2:**
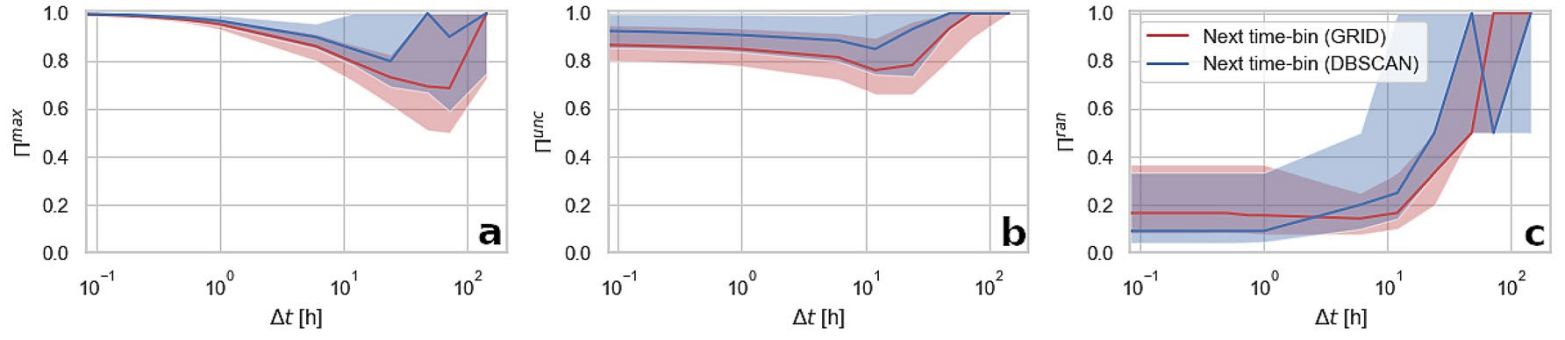
An impact of temporal resolution of data on the three types of predictability (actual (**a**), uncorrelated (**b**), and random (**c**) in the next time-bin sequences). The lines represent the median value and the shaded area indicate the interquartile range of predictability measures calculated for all the users in the dataset.

### Sequences properties and their relationship with predictability

Variations in predictability measures stem directly from changes in sequences of symbols introduced by different processing algorithms. To take a deeper insight into the impact of processing methods on the sequences themselves, we analyse them studying solely their geometrical and numerical properties.

#### Geometrical properties of sequences

**Figure 3:**
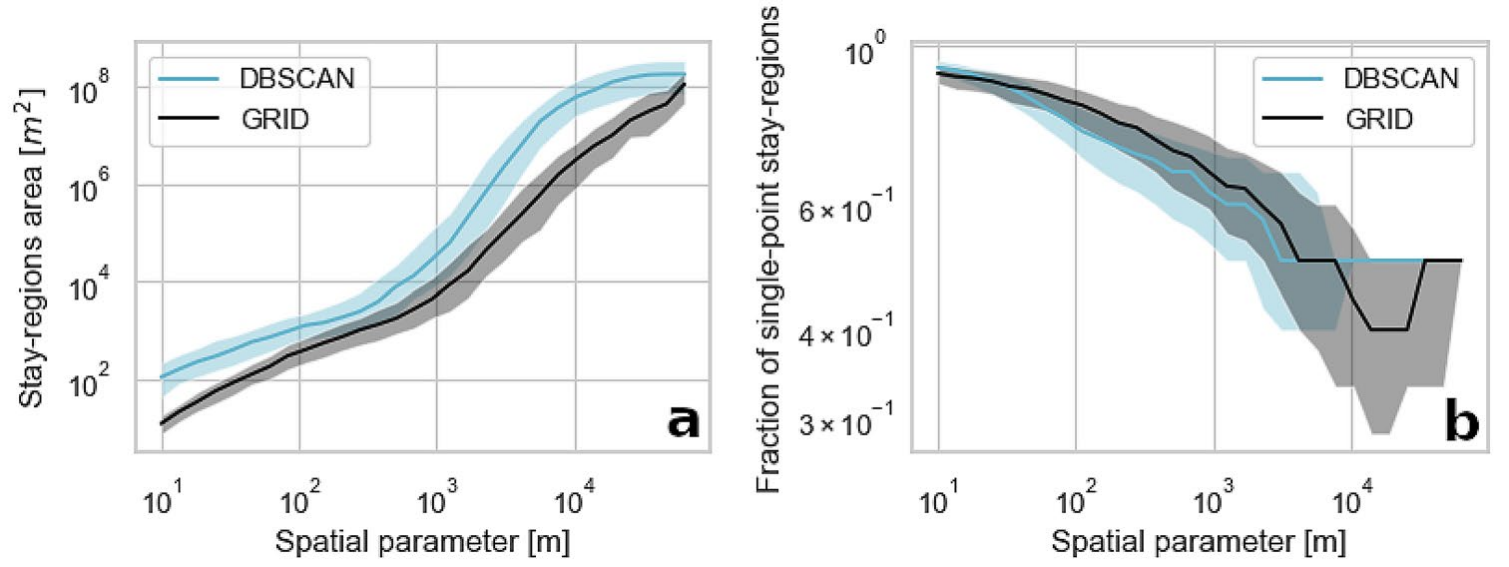
A dependence between the spatial aggregation method and the stay-regions area (**a**) and the fraction of single-point stay-regions (**b**). The lines represent the median value and the shaded area indicate the interquartile range of the measure calculated for all the stay-regions in the dataset. Spatial parameter relates to the variable controlling the magnitude of spatial aggregation in the grid-based approach (grid resolution) and the DBSCAN ($\epsilon$ parameter) method.

First, we look into the differences in stay-regions created through the grid-based and clustering spatial aggregation algorithms. As seen in Fig. 3(a), the size of stay-regions varies for the same values of controlling parameters (grid resolution and $\epsilon$ values) in the aggregation methods, thus they have a different sensitivity for their parameters. Clusters detected by the DBSCAN algorithm are usually spatially larger than data points groups aggregated in grid cells. However, the increase in an area of clusters created by the DBSCAN algorithm is lower for the lowest aggregation levels, which indicates the robustness of the clustering method in a stay-regions detection. The rapid growth of stay-regions areas, which occurs for parameters larger than few hundreds of metres, suggests that at this point stay-regions are merging, lowering the number of unique symbols in the sequences and thus, increasing the predictability. The data aggregation of the grid-based algorithm does not scale linearly with the increasing cell size.

Although studies suggest eliminating single-point stay-regions (stay-regions consisting of an only one stop) arguing their meaninglessness, we decide to analyse the fraction of such stay-regions in the overall number of detected stay-regions. Such locations are impossible to predict using conventional methods as they appear only once in the sequence. Although a person can visit a location only once over the course of a few days, Fig. 3(b) suggest that a grid-based algorithm tends to artificially separate stops from stay-regions resulting in a higher number of single-point stay-regions.

#### Numerical properties of sequences

**Figure 4:**
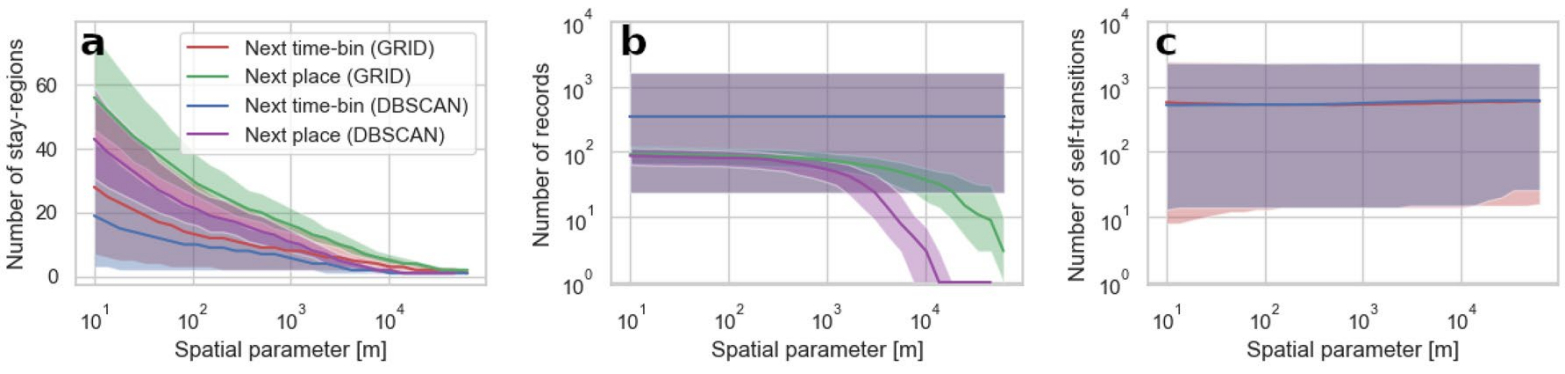
An impact of parameters controlling the spatial resolution of aggregated data on three types of quantitative properties of mobility sequences (number of stay-regions (**a**), number of records (**b**), and number of self-transitions (**c**)). The lines represent the median value and the shaded area indicate the interquartile range of the measure calculated for all the users in the dataset. Spatial parameter relates to the variable controlling the magnitude of spatial aggregation in the grid-based approach (grid resolution) and the DBSCAN ($\epsilon$ parameter) method.

**Figure 5:**
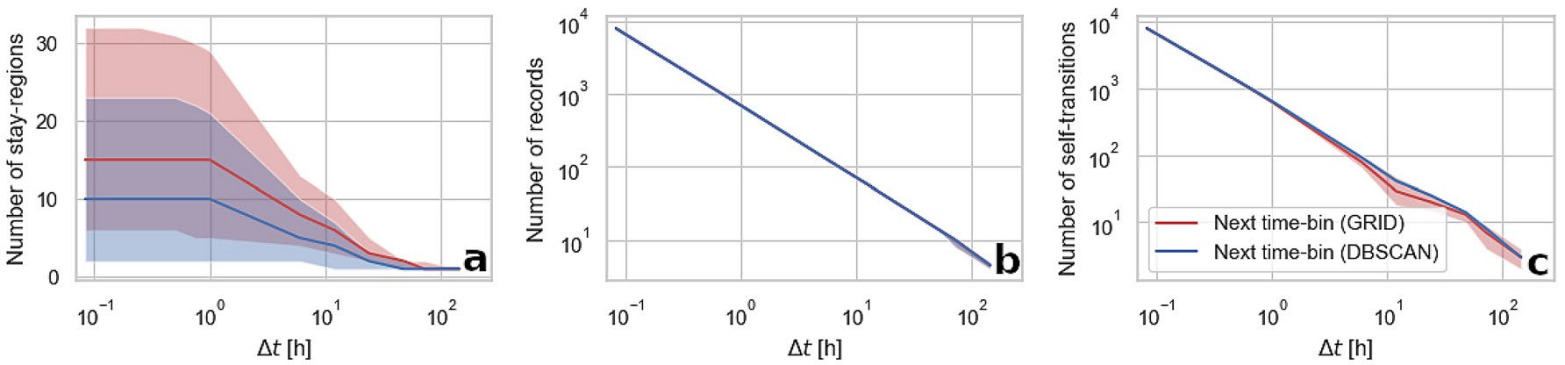
An impact of temporal resolution of data on three types of quantitative properties of the next time-bin sequences (number of stay-regions (**a**), number of records (**b**), and number of self-transitions (**c**)). The lines represent the median value and the shaded area indicate the interquartile range of the measure calculated for all the users in the dataset.

Impacts of the spatio-temporal granularity of the data on the quantitative properties of individual movement sequences are presented in Figs. 4 and 5. An increase in both, spatial and temporal aggregation, decrease the number of stay-regions present in sequences but for $\Delta t$ we find the number of stay-regions being stable up to 1-h resolution. In general, the number of detected stay-regions is higher for the next place sequences than for the next time-bin sequences. This effect is caused by the presence of short visits. The median time elapsed between starts of users’ visits in two distinct stay-regions is 167 min, while over 27% of stay-regions are visited for a period not exceeding 60 min. Therefore, in the next-time bin sequences, for higher levels of temporal aggregation, locations visited in a short time span are removed leaving only the one where a person stayed for the longest period, while in the next place sequences all these locations will be present. This effect aligns with the lower $\Pi _{max}$ of the next time-bin sequences as the higher number of stay-regions decreases their predictability because each stay-region is a unique symbol in the sequence.

Spatial aggregation methods, in general, do not affect the number of records and self-transitions in the next-time bin sequences. Their quantity is imposed only by their $\Delta t$ which present a logarithmic relationship ($R^2 > 0.99$). We find that the next time-bin sequences predictability is mainly affected by the number of self-transitions and that this number scales linearly with the sequence length. This effect is however stronger for sequences created with a clustering algorithm, especially for highly aggregated data where the linear relationship between the number of records and self-transitions in data aggregated into the grid is disturbed. In the case of the next place sequences, decreasing spatial resolution reduces the total number of records as stay-regions become larger and are eliminated. The effect of low records count is observable as the rapid changes of the next place sequences predictability (see Fig. 1).

## Discussion

We have adopted a wide variety of methods used in the last 11 years of research on human mobility predictability and applied them to a dataset of a high spatio-temporal resolution and completeness to understand how the data and used processing methods impact movement predictability. We confirm findings from previous works, finding entropy and predictability of movement sequences varying for different levels of spatio-temporal data resolution and processing techniques. In comparison to these works, we significantly extend the range of analysis, studying the impact of multiple spatial scales and a wide range of temporal resolutions, and transforming movement trajectories into two distinct types of movement sequences aggregated spatially with grid-based and clustering approaches. This enables us to fully estimate the relationship between predictability measures and processed sequences (see Figs. 1, 2).

We found that $\Pi ^{max}$ of the next time-bin sequences correlate negatively with a spatial resolution which aligns with findings from previous works^11,13–17^. We determined that this relationship is non-linear and close to exponential. On the other hand, an increase in $\Pi ^{max}$ observed in the data of high temporal resolution, which was identified in the literature, in our study was found to be limited to a certain level (in our case 1 and 6 days) where this dependency vanishes due to sequence shortening.

We have also studied the next place sequences which were previously proposed by Ikanovic and Mollgaard and Cuttone et al.^15,16^ but were not analysed for their predictability at various scales. We found the next place sequences being much less predictable than the next time-bin sequences, which also was identified in the aforementioned works, but only for a single algorithm setting. Interestingly, their $\Pi ^{max}$ grow slowly with a resolution decrease and at low temporal resolution rapidly changes due to the limited number of symbols in sequences. It is important to note that the Lempel-Ziv estimator converges to the actual entropy when the length of a sequence approaches infinity, hence, as noted by Teixeira et al.^14^, estimates for short next place sequences as well as low temporal resolutions are subject to error and should not be considered precise. This also explains why $\Pi ^{unc}$ for highly spatially aggregated data is higher than $\Pi ^{max}$.

The difference between the next time-bin and the next place sequences is in removing self-transitions from the latter. This procedure alters the predictability measure, which, instead of measuring the number of correctly predicted symbols in the whole sequence, is approximately measuring the number of correctly predicted transitions between locations, which from the perspective of mobility prediction are the most important. As previously noted^11,14,16^, a large number of self-transitions rapidly increases predictability as even a naive algorithm guessing that a person always stays in a previous location is achieving high prediction accuracy. Therefore, an increase in predictability for higher temporal resolutions is a result of a logarithmical growth in the number of self-transitions (see Fig. 5(c)). Spatial aggregation, however, in general, does not influence the number of self-transitions (see Fig. 4(c)), hence the increase in predictability together with the decrease of the spatial resolution of both types of sequences is most likely stemming from the lower number of extracted stay-regions. A decrease in the number of stay-regions can be linked to the number of containers, described in the recent work of Alessandretti, Aslak and Lehmann^24^, at different spatial scales within which data points can be grouped. Logically, it is easier to predict the location of an individual at the level of a country rather than at the level of buildings.

Previous works on human mobility predictability presented different ways to determine important locations from mobility data, which can generally be divided into the grid-based aggregation and clustering. Up till now, the impact of spatial data aggregation was evaluated using a grid of varying cell size. We presented and studied a commonly used representative of clustering methods, namely DBSCAN, and compared it to the results obtained via grid aggregation. We found data processed with DBSCAN to be more predictable at all levels of aggregation. The reason for that is the grid-based aggregation introducing additional uncertainty into the data by detecting more single-point stay-regions than DBSCAN (see Fig. 3(b)). Stay-regions may have different sizes and are unevenly spread in space, therefore it is not possible to select a grid cell size that would correspond to all detected locations. It has been identified that grid aggregation can split neighbouring data points^29^. DBSCAN tends to create larger clusters than the grid-based algorithm for the same values of a parameters controlling the magnitude of spatial aggregation. However, the DBSCAN $\epsilon$ parameter cannot be directly related to the spatio-temporal magnitude of aggregation, as a spatio-temporal resolution of outcoming data depends also on the topology of data points.

We have also compared the entropy estimation methods for the next time-bin sequences with missing data. Among three proposed methodologies^5,15,17^, the method denoted as $\hat{H}_{\Delta e}$ was performing best. However, it is important to note that this approach requires some representative trajectories in a studied dataset to have a low fraction of missing data ($q < 0.25$) which significantly limits the applicability of this approach. Although, we were able to apply this method on our dataset, reported in the related literature values of completeness are $q \in [0.7;0.9]$ for mobile phone data and $q \in [0.2;0.9]$ for GNSS data. We found errors of $\hat{H}_{shuff}$ and $\hat{H}_{unc}$ methods to be relatively high, especially for high temporal resolutions. A functional dependence between *q* and estimated entropy in the $\hat{H}_{shuff}$ method varies between linear and exponential, while in the $\hat{H}_{unc}$ it is always exponential, making it easier to fit and extrapolate a function, which in turn results in overall higher accuracy. Because our dataset had a low fraction of missing data and all movement trajectories had $q<0.15$, we did not have to use any of the estimation methods, thus their errors did not influence obtained results.

Our study has limitations. First of all, although we used a sample of human mobility data of high spatio-temporal granularity, the impact of data processing may be different for another area as mobility can be influenced by factors such as weather conditions and climate^30^. Secondly, we did not fully study why the predictability of mobility sequences is altered at specific spatio-temporal scales. We found, for example, that not all increases in the predictability of the next time-bin sequences can be explained by the increase in the number of self-transitions. Therefore, there is a need for further in-depth investigation of properties of mobility sequences to explain what and how changes introduced into the mobility sequences influence their predictability. The ultimate goal of further research is not only to explain these dependencies but to indicate how mobility data should be processed to maximise retained information, avoid bias, and increase their utility.

In summary, to the best of our knowledge, this paper is the first to study the impact of spatio-temporal resolution and data processing algorithms on the predictability and associated properties of movement trajectories to such an extent. We analysed how the measure of predictability change, reaching extremely high and low spatio-temporal scales and attempted to explain the functional dependence between these values. We applied approaches commonly used in the literature to process human mobility data, presenting their impact on the created movement sequences and discussing their advantages and disadvantages. This work is a step towards understanding how the processing of individual movement trajectories, which is directly related to the way of their representation, influences the obtained results.

## Methods

### Stationary points detection

To estimate the impact of the spatio-temporal resolution of data on predictability, the movement trajectories of individuals have to be processed. First, we apply a stationary points detection algorithm^31^, which is common for all the evaluated processing methods. Let the movement trajectory $P_i = \{p_1, p_2,\ldots ,p_{T_i}\}$ be a sequence of $T_i$ data points recorded by a GNSS device *i* representing the movement of an individual. Each data point is a triplet $(x_{T_i},y_{T_i},t_{T_i})$, where $x_{T_i}$ and $y_{T_i}$ are coordinates recorded at time $t_{T_i}$. The method accepts distance $\delta$ and time $\tau$ thresholds as parameters. The goal is to extract a sequence of *n* stay-points $S_i = \{s_1, s_2,\ldots ,s_n\}$ for each recorded movement trajectory. Each stay-point is is a quadruplet $(x_n,y_n,start_n,end_n)$, where $x_n$ and $y_n$ coordinates are a centre of a stay-point which was visited between $start_n$ and $end_n$ time. The algorithm scans through a movement trajectory $P_i$ in a temporally ascending order, starting from the first point that is not yet assigned to any stay-point. Then, it iterates through the consecutive points, at each step calculating a distance between the current and the first point from which stay-point detection has started. If that distance is lower than $\delta$, the current data point is accumulated to a single stay-point, together with all previous records. When the distance is larger than $\delta$, the algorithm checks the time interval between the first and the last of data points assigned to the currently processed stay-point. If it is larger than $\tau$, data points are selected as a stay-point, otherwise, all points are discarded. After that, the algorithm starts searching for the next stay-point starting from the fist data point which failed to be within the $\delta$ threshold and the process repeats again. Following previous works^19^, we set $\delta$ = 300 metres and $\tau$ = 10 min to detect stay-points in our dataset.

### Stay-regions detection

To study the impact of data resolution on predictability, we aggregate data spatially and temporally. First, we execute the spatial aggregation where stay-points are aggregated from a sequence $S_i$ into sequence of stay-regions $X_i = \{x_1,x_2,\ldots ,x_{L_i}\}$, so each stay-region consist of spatially close stay-points. In practice, we create a dictionary where each stay-point is assigned to a stay-region and a stay-region can consist of multiple stay-points. We then use that dictionary to map a sequence $S_i$ into a sequence of stay-regions $X_i$. It is important to precede temporal aggregation with spatial aggregation, as the reversed process could result in data loss. For spatial aggregation, we use two commonly used methods, that is grid-based aggregation and clustering.

#### Grid-based aggregation

For the grid-based aggregation, we generate a regular grid of cells of size *G*, where *G* is the spacing between grid nodes. The grid covers the spatial extent of the stay-points and divides the area into regular grid cells. Stay-points lying within a cell are assigned to its centre. For *G* values we use a set of 30 values (to provide enough insight simultaneously limiting the computational time) spaced evenly on a log scale, starting from $10^1$ to $10^{4.8}$, where the maximum value corresponds approximately to a half of the dataset’s maximum spatial extent. We decided to use a log scale because we expect predictability to be more vulnerable to changes at high spatial resolutions.

#### DBSCAN

DBSCAN iterates through each stay-point and assigns it to a cluster (stay-region) if a distance from any stay-point in the cluster is smaller than the distance defined by the $\epsilon$ parameter. Therefore, to analyse how movement sequences change for varied levels of spatial aggregation, we vary the $\epsilon$ parameter, testing 30 values evenly spaced on a log scale, starting from $10^1$ to $10^{4.8}$. The cluster has to consist of at least one point to be considered as meaningful.

### Next time-bin and next place sequences

We process movement trajectories from detected stay regions $X_i$ into the next time-bin and next place sequences.

#### Next time-bin sequences

For the next time-bin sequences, $X_i$ of each person is transformed into a vector of evenly spaced on a time scale time-bins, where each time-bin has size $\Delta t$. For each time-bin, a current position is recorded as $x_n$ visited during this particular time-bin. In situations when in the same time-bin, more than one place was visited, the one where the person spent more time is selected. If locations have the same visiting time in the same time-bin, then the one which was more often visited is selected. If no location was visited during the time-bin, a null value is assigned to the vector, creating missing data. In this study, to simulate various data temporal resolution, we select different $\Delta t$ values to create the next time-bin sequences. These values are 5, 10, 15, 30, 45, 60 min and 1, 6, 12, 24, 48, 72, 144 h.

#### Next place sequences

The next place sequences are designed to represent transitions between locations only. They are created by removing all consecutively repeating stay-regions from $X_i$ of each person.

### Entropy and predictability measures

Following the previous works^5,12^, we calculate the three types of entropy. The random entropy, $S^{rand}_i = \log _2L_i$, measures sequence uncertainty assuming that each location in a sequence is visited with the same frequency. Predictability calculated on $S^{rand}_i$ assumes that only the number of unique symbols in the sequence is known and represents sequence predictability which can be reached randomly guessing symbols in the sequence. The uncorrelated entropy, $S^{unc}_i = -\sum ^{L_i}_{k=1}p_k\log _2p_k$, assumes that visitation frequency for each stay-region is known and is denoted as $p_k$. Predictability associated with this entropy corresponds to an accuracy which can be reached by drawing symbols from the known frequency distribution. The actual entropy, $S_i = -\sum _{X^{'}_{i} \subset X_{i}} P(X{'}_{i})\log _2[P(X{'}_{i})]$, where $P(X{'}_{i})$ is the probability of finding a particular time-ordered subsequence $X{'}_{i}$ in the $X_i$ sequence. Predictability associated with the actual entropy represents the theoretical upper bound of predictability of the sequence, capturing the full spatiotemporal order of data points in it. Because the direct computation of probability $P(X^{'}_{i})$ is highly time-consuming, Song et al.^5^ proposed to estimate the actual entropy using the Lempel-Ziv compression algorithm given as $S^{est} = (\frac{1}{n}\sum _j \Lambda _j)^{-1}\log _2 n$, where $\Lambda _j$ denotes the length of the shortest substring starting at position *j* of the sequence, which does not appear from position 1 to $j-1$. Such estimated entropy converges to the actual entropy when *n* reaches infinity. Importantly, when a unique substring cannot be found, then $\Lambda _j = n - j + 2$ for all remaining positions^32^.

The upper limit for the predictability can be calculated from the derived entropies by solving Fano’s inequality, which is $\Pi _i \le \Pi ^{Fano}_i (E,L_i)$. $\Pi ^{Fano}_i$ is given by $E = -\Pi ^{Fano}_i\log _2(\Pi ^{Fano}_i)-(1-\Pi ^{Fano}_i)\log _2(1-\Pi ^{Fano}_i) + (1-\Pi ^{Fano}_i)\log _2(L_i-1)$. When we substitute *E* by $S^{rand}_i$, $S^{unc}_i$, or $S^{est}_i$ we are able to calculate random, uncorrelated and actual predictability, respectively. It is important to note that the logarithm base in Fano’s inequality has to be the same as the ones used to estimate entropies^32^.

### Estimating actual entropy from missing data

When the next time-bin sequences are created, some of the time-bins may be empty, which artificially increases the sequence’s entropy^5^. The ratio of missing time-bins is denoted as *q*. We compare three distinct approaches for estimating actual entropy from sequences with missing data, which were proposed in the literature. For the experiment, we calculate a reference value of actual entropy *H* from 100 trajectories with $q < 0.15$, which are considered to present true entropy. We artificially remove portions of data from them to simulate various levels of *q*. Specifically, these are $q = 0.15, 0.20, 0.25,\ldots , 0.60$. Then for each level, we estimate the actual entropy using three different methods and calculate the error as $\hat{H}/H$, where $\hat{H}$ is an estimated entropy and *H* is a true entropy calculated for the complete sequence.

Song et al.^5^ proposed an algorithm which we denote as $\hat{H}_{shuff}$. Given a sequence, the algorithm increases its *q* to $q' = q + \Delta q$, where $\Delta q = 0.00, 0.05, 0.10,\ldots ,0.90-q$ and for each $q'$ calculates the order parameter $\sigma (q') = \log _2(S^{est}(q'/S^{unc}(q'))$, where $S^{est}(q')$ is estimated using the Lempel-Ziv algorithm and $S^{unc}(q')$ is determined using the Lempel-Ziv algorithm on the same sequence which is randomly shuffled. This enables calculation of a series of order parameters for different levels of $q'$, which are then extrapolated to $q' = 0$, giving a $\sigma _{est}$ at $q = 0$. The entropy is calculated as $\hat{H}_{shuff} = 2^{\sigma _{est}} S^{unc}(q)$, where $S^{unc}(q)$ is calculated using the Lempel-Ziv algorithm over the randomly shuffled input sequence.

Ikanovic et al.^15^ proposed a very similar algorithm, but instead of calculating $S^{unc}(q')$ with Lempel-Ziv algorithm over the randomly shuffled sequence, they proposed to use equation for uncorrelated entropy, that is $S^{unc}(q') = -\sum ^{L_i}_{k=1}p_k\log _2p_k$, as a scaling feature. The rest of the algorithm remains identical. We denote this method by $\hat{H}_{unc}$.

Another algorithm was proposed by Lin et al.^17^. Similarly to previously presented approaches, the *q* of the sequence is increased to $q' = q + \Delta q$. Then, for each $q'$ an error $\Delta e = \frac{\hat{H}(q') - \hat{H}(q)}{\hat{H}(q)}$ is calculated, where $\hat{H}(q')$ is an entropy calculated using the Lempel-Ziv algorithm for a sequence with $q'$ fraction of missing data. Calculated errors $\Delta e$ are used to estimate the average entropy estimation error for various levels of $q'$. These estimations are then used to correct entropies estimated from sequences with larger *q*. We denote this method as $\hat{H}_{\Delta e}$. It is important to note that movement trajectories used to estimate $\Delta e$ in our experiment are not used in the evaluation, as this would artificially decrease an estimation error.

### Ethics approval and consent to participate

We hereby confirm that ethics approval for data collection and research in this study was received from the Institute of Geodesy and Geoinformatics Human Mobility Ethics Committee. All participants of the study have been acknowledged with the data collection process and delivered informed consent to use data collected from them, confirming that they understand what kind of data will be collected from them and that they can withdraw from the study at any moment. We confirm that all the research methods were carried out in accordance with relevant guidelines and regulations.

## Data Availability

The datasets of statistical measures calculated and analysed during the current study are available in the Zenodo repository, 10.5281/zenodo.4893432. The code produced during this study is a part of a HuMobi programming library and is available at the GitHub repository, 10.5281/zenodo.4893369.
